# Xenotropic and Other Murine Leukemia Virus-Related Viruses in Humans

**DOI:** 10.1155/2011/787394

**Published:** 2011-12-20

**Authors:** Arifa S. Khan, Myra McClure, Yoshinao Kubo, Paul Jolicoeur

**Affiliations:** ^1^Laboratory of Retroviruses, Division of Viral Products, Center for Biologics Research and Review, US Food and Drug Administration, Bethesda, MD 20892, USA; ^2^Jefferiss Research Trust Laboratories, Wright-Fleming Institute, Faculty of Medicine, Imperial College London, Norfolk, London W2 1PG, UK; ^3^Department of AIDS Research, Institute of Tropical Medicine, Nagasaki University, Nagasaki 852-8523, Japan; ^4^Laboratory of Molecular Biology, Clinical Research Institute of Montreal, Qc, Canada H2W 1R7; ^5^Department of Microbiology and Immunology, Université de Montréal, Montreal, Qc, Canada H3C 3J7; ^6^Division of Experimental Medicine, McGill University, Montreal, Qc, Canada H3A1A3

The recent discovery of a xenotropic murine leukemia virus-related retrovirus (designated as XMRV) in prostate cancer tissues and later in chronic fatigue syndrome (CFS) patients created excitement related to possible association of a virus with these human diseases. However, the failure to reproduce such results in other laboratories raised concerns and much debate among scientists, patient populations, and clinicians over the original findings. This special issue is a collection of recent research reports, that address this controversy. It also includes several expert reviews on various aspects of murine retroviruses, such as virus biology, replication, phylogeny, and pathogenesis, as well as XMRV in prostate cancer. 

The *first* paper by A. Rein reviews the murine retrovirus genomic structure, viral structural proteins, and virus replication. The *second* paper by C. A. Kozak provides a detailed review of murine retrovirus entry into the cell and different receptor usage by different types of murine retroviruses with comparison to XMRV. The *third* paper by J. Blomberg et al. discusses the phylogenetic analysis of murine retroviruses and other retroviruses. The *fourth* paper by J. Chakraborty et al. discusses murine retrovirus pathogenesis and a mouse model for transmission of lymphoma by breast milk. The *fifth* paper by D. E. Kang et al. reviews the discovery, progress, and current status of XMRV findings in prostate cancer patients. The *sixth* paper by S. Tang and I. K. Hewlett reviews XMRV detection assays and deficiencies in the testing methods. The *seventh* paper by J. M. Coffin and O. Cingöz provides a detailed review of the controversies related to the XMRV results in human clinical samples and the findings regarding virus origin and discusses the potential sources of contamination that resulted in the misidentification of the virus as a novel human retrovirus. 

Additionally, the special issue contains various research papers demonstrating the absence of XMRV in various patient populations using sensitive assays for virus detection. The *eighth* paper by B. Oakes et al. reports the absence of antibodies in CFS patients and healthy controls using two novel sensitive immunoassays. The *ninth* paper by J. Spindler et al. reports the lack of evidence of XMRV infection in HIV-1 infected men or men at high risk for HIV-1 infection by analyzing PBMCs and plasma samples using sensitive PCR assays and immunoassays. The *tenth* paper by K. A. Delviks-Frankenberry et al. demonstrates the absence of XMRV in PBMCs and plasma from HIV-1 lymphoma patients using PCR or immunoassays. The *eleventh* paper by M. J. Robinson et al. indicates the absence of XMRV sequences in prostate cancer samples from diverse populations, B-cell lymphoma patients, as well as UK blood donors. The *twelfth* paper by M. Kearney et al. reports the use of different methodologies to demonstrate the absence of XMRV in plasma and in some tissue samples from prostate cancer patients. The final, *thirteenth* paper by P. Sharma et al. describes XMRV infection in the reproductive tissues of rhesus monkeys, proposing the possibility of an animal model for further investigations of virus transmission.

This special issue provides the recent thinking and research results of studies on XMRV and other murine leukemia retrovirus-related sequences in humans. Numerous studies have failed to confirm the presence of XMRV in humans, and XMRV has recently been found to be a laboratory-derived rare recombinant, which originated by xenografting a patient's prostate cancer cells in *nude* mice. A consensus has now emerged that XMRV footprints or infectious XMRV detected in normal human individuals or in some diseased patients represents laboratory contaminations. The information provided in this issue should be of interest to a broad audience including scientists, clinicians, patient populations, and public health agencies. 

*Arifa S. Khan* *Myra McClure* *Yoshinao Kubo* *Paul Jolicoeur*

